# Imputing unjustified bulk density values to soils with biochar addition biases soil carbon sequestration estimates

**DOI:** 10.1073/pnas.2528257123

**Published:** 2026-01-07

**Authors:** Daquan Sun

**Affiliations:** ^a^Institute of Soil Biology and Biogeochemistry, Biology Centre, Czech Academy of Sciences, České Budějovice CZ-37005, Czech Republic

Yang et al. analyzed a global dataset including 29 long-term field experiments (4 to 12 y) and reported sustained benefits of biochar for crop yield, greenhouse gas mitigation, and soil organic carbon (SOC) sequestration (1). SOC density (SOCD, kg C ha^−1^) was estimated as:

[1] SOCD=SOC×BD×H×10,

where SOC is the SOC content (g kg^−1^) of the plow horizon, BD is the soil bulk density (g cm^−3^), and H is the depth of the plow horizon (0.2 m).

**Fig. 1.**
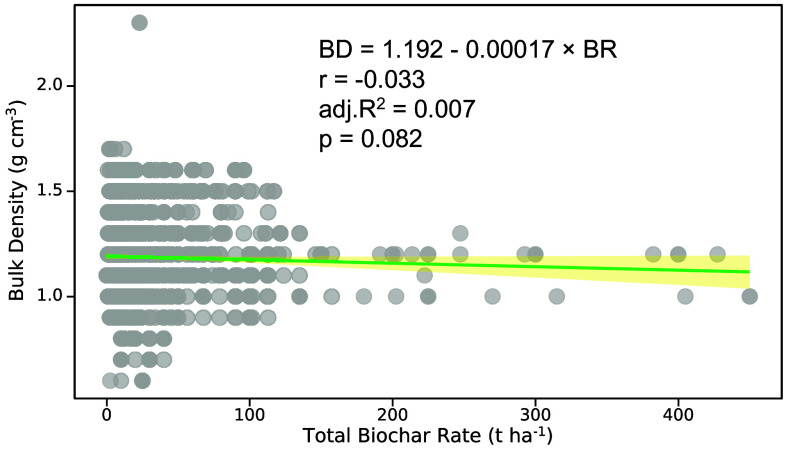
Soil bulk density (g cm^−3^) correlation with total biochar application rate (t ha^−1^) using the data in the supplemental table from Yang et al.’s study (1). BD: bulk density; BR: biochar application rate.

However, in the supplemental dataset of Yang et al. (1), soils receiving high biochar application rates were frequently assigned the same BD (g cm^−3^) as soils with low biochar addition. This contradicts well-established empirical evidence that BD (g cm^−3^) decreases as biochar application increases because biochar is substantially less dense than most soils (2, 3). In addition, numerous BD (g cm^−3^) values in the supplemental dataset appear to have been imputed without regard to parental soil texture (loam, clay, sandy), which is a primary determinant of soil BD (4). As a consequence, BD (g cm^−3^) shows no significant correlation with biochar application rate in the dataset (r = –0.033, adj. R^2^ = 0.0007, *P* = 0.082), a result highly unlikely if BD (g cm^−3^) had been consistently and accurately reported or imputed (Fig. 1).

Because BD (g cm^−3^) is directly included in the SOCD Eq. **1**, disregarding its dependence on biochar application rate and parental soil texture leads to systematic overestimation of SOC stocks.

To correctly account for the effect of biochar on the mixture bulk density of soil (BD_mix_) using and biochar BD (BD_B_) and soil BD (BD_s_) can be expressed as

[2] $${\mathrm{BD}}_{\mathrm{mix}}=\frac{1+C}{\frac{C}{{\mathrm{BD}}_{B}}+\frac{1}{{\mathrm{BD}}_{S}}},$$

where C = m_B_/m_S_ is the biochar-to-soil mass ratio. C can be calculated directly from the biochar application rate:

[3] $$C=\frac{{\mathrm{Rate}}_{\mathrm{BC}}}{{\mathrm{BD}}_{S}\cdot H\cdot 10,000},$$

where Rate_BC_ is the biochar application rate (t ha^−1^) and H is plow depth (m) such as 0.2 m as used in Yang et al. (1).

To quantify the extent of BD and SOCD overestimation, I reexamined the dataset in Yang et al. (1) by selecting soil BD (g cm^−3^), total biochar rate (ton ha^−1^), and soil texture, after removing entries with missing values. Soil BD (BD_s_) was assigned based on texture: loam (1.25 g cm^−3^) (2), sandy (1.76 g cm^−3^) (2), and clay (0.78 g cm^−3^) (4). Biochar BD (BD_B_) was taken as 0.33, 0.24, and 0.22 g cm^−3^, respectively (2). After recalculating C and BD_mix_ using Eqs. **2** and **3**, BD_mix_ values were approximately 3 to 5 times lower (i.e., one-third to one-fifth as large) than the soil BD (g cm^−3^) values used in Yang et al. (1). Because SOCD is proportional to BD (Eq. **1**), this indicates a comparable overestimation of SOC sequestration and the associated climate mitigation benefits in the original analysis, if not incorporating these corrections.
